# Management and clinical outcomes of follicular lymphoma across continuous lines of treatments: a retrospective analysis in China

**DOI:** 10.3389/fonc.2023.1264723

**Published:** 2023-10-24

**Authors:** Jiaxin Liu, Yunfei Hu, Linjun Zhao, Reyizha Nuersulitan, Yanfei Liu, Hui Yu, Yingying Ye, Dedao Wang, Yongjing Tang, Feier Feng, Weiping Liu, Jun Zhu, Lingyan Ping, Yuqin Song

**Affiliations:** ^1^ Key Laboratory of Carcinogenesis and Translational Research (Ministry of Education/Beijing), Department of Lymphoma, Peking University Cancer Hospital & Institute, Beijing, China; ^2^ Department of Oncology, Guizhou Medical University and Department of Lymphoma, Affiliated Hospital of Guizhou Medical University, Affiliated Cancer Hospital of Guizhou Medical University, Guiyang, China; ^3^ Peking University International Hospital & Institute, Department of Lymphoma, Beijing, China

**Keywords:** relapsed/refractory, follicular lymphoma, Chinese, treatment patterns, prognosis, POD24

## Abstract

**Background:**

Follicular lymphoma (FL) is characterized by an incurable course that frequently necessitates multiple lines of treatment. While a range of new approaches have broadened therapeutic options for patients in later lines, data regarding treatment patterns and outcomes of Chinese patients with relapsed/refractory(R/R) FL was scarcely reported.

**Methods:**

This retrospective single-center study included patients diagnosed with FL grades 1–3a at our institution between January 2002 and December 2019. Endpoints of interest were analyzed according to lines and types of interventions. The endpoints mainly included overall response rate (ORR), progression-free survival (PFS), and overall survival (OS).

**Results:**

The study enrolled 566 biopsy-proven patients. Among them, 544 patients initiated the first line of treatment, followed by 240 initiating the second line, 146 initiating the third line, 88 initiating the fourth line, 47 initiating the fifth line, and 28 initiating the sixth line. In terms of treatment patterns, anti-CD20 chemotherapy was a major modality in the first and second lines. However, for patients in the third line and subsequent lines, treatment approaches were diverse, and participation in clinical trials for new medications was common, which correlated with a survival benefit. The study also revealed that clinical indicators (such as ORR, PFS, and OS) gradually decreased with each subsequent line of treatment. The ORR at the first line was 86.6%, but decreased to 48.6% at the third line and 40.4% at the sixth line, respectively. Similarly, median OS and PFS decreased to 88.8 and 7.1 months at the third line and further reduced to 21.7 and 2.8 months at the sixth line, respectively. A total of 133 patients developed progression within 24 months from the initiation of first line anti-CD20 chemotherapy (POD24), and these patients exhibited poorer response rates and outcomes in subsequent lines of therapycompared to the non-POD24 group.

**Conclusion:**

This study revealed the clinical routine practices and prognosis of R/R FL patients within the Chinese population. It underscored the unmet need for optimal strategies to improve survival and also served as a benchmark for future trials.

## Introduction

The burden of lymphoma has significantly increased in non-Hodgkin lymphoma over the past three decades (1). Among indolent non-Hodgkin lymphoma (iNHL), follicular lymphoma (FL) is the most common subtype (2). An analysis reported a 10-year overall survival (OS) of 80% among the populations in the USA in the rituximab era (3). The combination of anti-CD20 antibody and chemotherapy, known as immunochemotherapy (IM), has been recommended as the first-line treatment for advanced-stage FL patients with grades 1–3a. This regimen has demonstrated an overall response rate (ORR) of approximately 90% and a median progression-free survival (PFS) ranging from 31 to 69 months (3–5). However, approximately 20% of patients experience disease progression within 24 months (POD24) from the initiation of first-line IM. Notably, POD24 is strongly associated with a worse prognosis for FL patients; the 5-year OS rate was approximately 50% in patients with POD24 compared to 90% in patients without POD24 (6, 7). Furthermore, a majority of FL patients continue to endure multiple relapses, leading to a decline in both the duration of remission and health-related quality of life (HRQoL) with each subsequent treatment session (8, 9). Therefore, there is a growing need to improve the prognosis of these patients.

When FL patients experienced relapsed/refractory (R/R) events and required treatment, the usual options contained chemotherapy alone, anti-CD20 antibody alone, IM, radiotherapy, and stem-cell transplantation (SCT), including autologous and allogeneic SCT (auto-SCT and allo-SCT). Recently, several novel approaches for R/R FL have emerged, including immunomodulatory drugs, targeted small-molecule inhibitors, bispecific antibodies, chimeric antigen receptor T (CAR T)-cell therapy, etc. (5). Remarkably, CAR T-cell therapy has shown impressive clinical responses and holds great promise in B-cell lymphoma (10–12). Lenalidomide, an immunomodulatory drug, has demonstrated a significant anti-tumor effect when combined with rituximab (R2 regimens) in R/R FL patients (13). Meanwhile, phosphoinositide 3-kinase (PI3K) inhibitors initially received approval for the treatment of R/R FL. However, all have been withdrawn afterward, with the exception of copanlisib, due to unbearable adverse events (5, 14). In addition, enhancer of zeste homolog 2 specific (EZH2) inhibitor, another kind of small-molecule agent, has shown effectiveness in R/R FL patients with both mutant-type and wild-type EZH2 mutations, although the clinical efficacy appears to be better in the former group (15, 16). While B-cell leukemia/lymphoma-2 (BCL-2) overexpression is required for the pathogenesis of FL, the efficacy of BCL-2 inhibitor venetoclax as monotherapy was suboptimal (17). Similarly, Bruton’s tyrosine kinase (BTK) inhibitor ibrutinib in R/R FL patients has shown a response rate of only 21% when administrated as a single-agent application (18). The spleen tyrosine kinase (SYK) inhibitor has demonstrated limited efficacy for B-cell malignancies (19).

In the current situation, with numerous options available, determining the optimal approach for R/R FL patients remains an unmet need. Moreover, data regarding long-term survival and recurrence patterns across successive lines are relatively limited in China. Concurrently, clinical trials have rapidly evolved and emerged as a crucial alternative for patients in later lines of treatment. Nevertheless, as clinical trials typically follow a single-armed design, there is a lack of comparative data with patients receiving standard care. Therefore, the objective of this study was to explore the clinical characteristics, treatment patterns, and outcomes of FL patients in the modern treatment era.

## Materials and methods

### Patients and data collection

In this retrospective, single-center study, medical records of patients aged ≥18 years and diagnosed with FL grades 1–3a from January 2002 to December 2019 in Peking University Cancer Hospital were reviewed. The exclusion criteria were as follows (1): grade 3b FL or biopsy-proven histopathological transformation at diagnosis and (2) those who lacked treatment information and lost to follow-up. Data, including clinical features and medical interventions, were obtained through reviews of hospitalized and out-patient records; extended follow-ups were performed via outpatient review or telephone. Watch and wait (WW) was defined as the initiation of treatment performed at least 6 months after the diagnosis. The number of treatment lines was defined as the number of systemic therapies received after diagnosis. Rituximab maintenance and transplantation as consolidation were not counted as an additional line of therapy. This study has been approved by the independent ethics committee of our institution.

### Assessment and statistical methods

The endpoints of interest were overall response rate, progression-free survival, and overall survival. The ORR was defined as the proportion of patients who achieved a complete response or partial response. PFS at each line was defined as the time from initiation of each line of treatment to progression, relapse, death, or the date of last follow-up. Overall survival (OS) was defined as the time from diagnosis until death from any cause or date of last follow-up. The OS at each line was defined as the time from initiation of each line of treatment until death from any cause or date of last follow-up. Other endpoints of interest were the complete response (CR) rate, defined as the proportion of patients achieving CR, and time to the next line of treatment (TTNLT, defined as the time from initiation of treatment to initiation of the next line of treatment). Responses were assessed using Lugano 2014 criteria by positron emission tomography (PET) computed tomography (CT) scans, or both. POD24 was defined as progression within 24 months from the initiation of first-line anti-CD20 in conjunction with chemotherapy.

Continuous variables were described as means with ranges, and categorical variables were described as numbers and percentages. A comparison of different treatment responses was evaluated by Chi-square test. Kaplan–Meier method was used for the analysis of time-to-event outcomes, and differences were assessed using the log-rank test. A two-tailed *p* < 0.05 was considered statistically significant. The data were analyzed using SPSS Statistics 25.0 software. The Sankey diagram and survival curves across continuous lines of treatments were prepared in R language.

## Results

### Baseline characteristics

**Figure 1:**
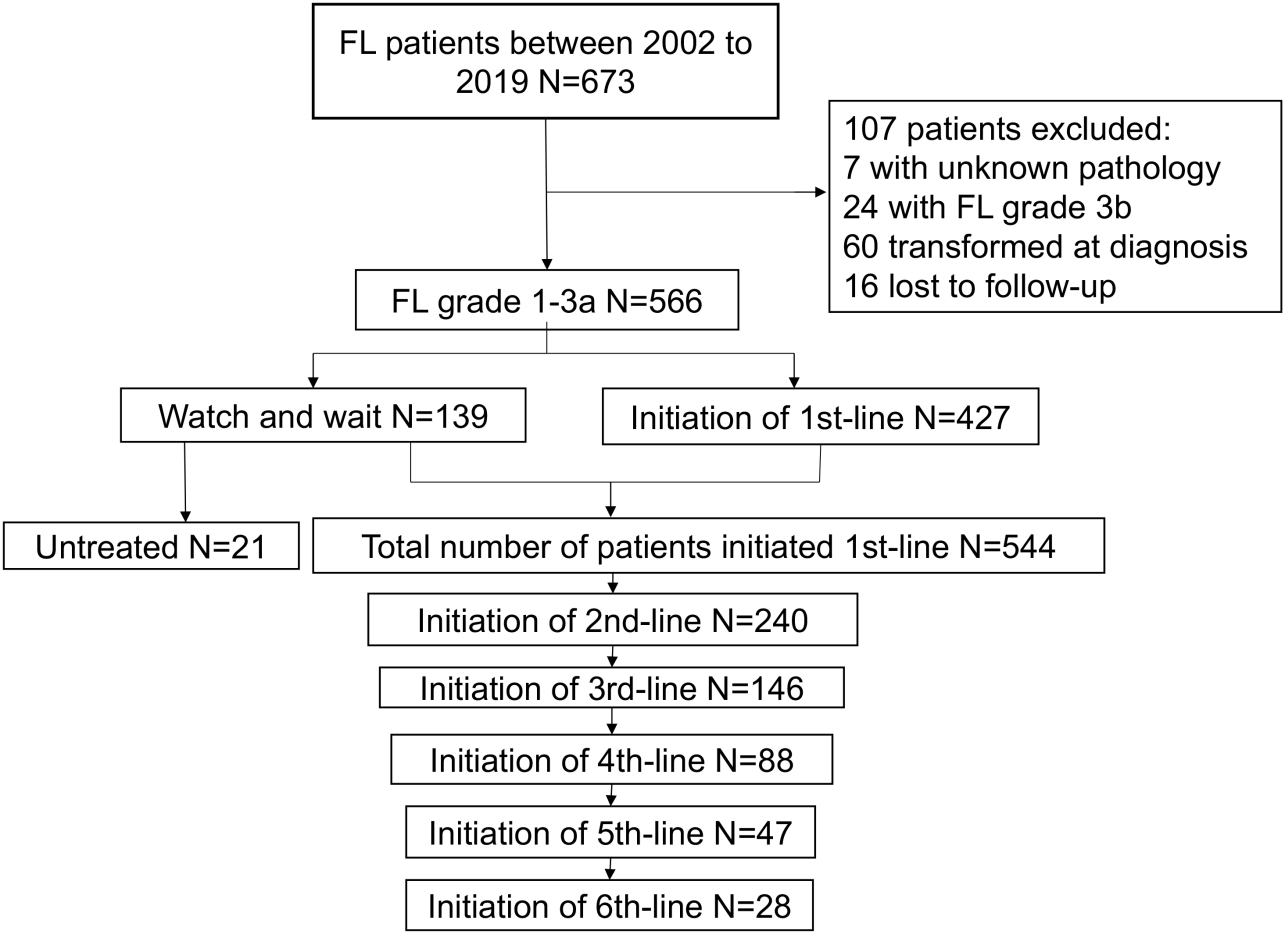
Study flow of patient enrollment in our cohort.

A total of 673 patients were originally identified, of whom 107 patients were excluded; finally, 566 patients were included for analysis. Among them, 139 patients chose WW, with 21 remaining WW until the last follow-up, while 118 initiated first-line therapy. A total of 544 patients received first-line treatment. During the follow-up, patients who received second-line, third-line, fourth-line, fifth-line, and sixth-line treatments decreased from 240 to 146, 88, 47, and 28, respectively (**Figure 1**). Baseline characteristics at diagnosis and treatment initiation are shown in **Table 1**. The median age at diagnosis and treatment initiation was 49 (range: 23–86) and 50 (range: 23–86) years, respectively. The proportion of patients with high-risk factors such as anemia at treatment initiation was higher compared to that at diagnosis. Among 544 patients initiating first-line treatment, 324(59.6%) patients reported GELF criteria.

**Table 1:** Patient characteristics at diagnosis and treatment initiation.

Clinical features	Diagnosis (*n* = 565)	Treatment (*n* = 544)
	*n* (%)	*n* (%)
Median age	49 (23–86)	50 (23–86)
Age ≤ 60	470 (83.2%)	447 (82.2%)
Age > 60	94 (16.8%)	97 (17.8%)
Sex		
Male	267 (47.3%)	256 (47.1%)
Female	298 (52.7%)	288 (52.9%)
Stage		
I–II	105 (18.6%)	89 (16.4%)
III–IV	457 (80.9%)	451 (82.8%)
Unknown	3 (0.5%)	4 (0.8%)
Histology grade		
FL grades 1–2	461 (81.6%)	443 (81.4%)
FL grade 3a	96 (17.0%)	93 (17.2%)
Unknown	8 (1.4%)	8 (1.4%)
ECOG PS		
0–1	515 (91.1%)	487 (89.5%)
≥2	28 (5.0%)	35 (6.5%)
Unknown	22 (3.9%)	22 (4.0%)
Nodal involvement		
≤ 4	182 (32.2%)	158 (29.3%)
>4	378 (66.9%)	382 (70.2%)
Unknown	5 (0.9%)	4 (0.7%)
B symptoms		
No	443 (78.4%)	420 (77.2%)
Yes	98 (17.3%)	107 (19.7%)
Unknown	24 (4.2%)	17 (3.1%)
Bone marrow involvement		
No	345 (61.1%)	304 (55.9%)
Yes	216 (38.2%)	235 (43.2%)
Unknown	4 (0.7%)	5 (0.9%)
Hemoglobin (g/dL)		
≥12	395 (70.0%)	377 (69.3%)
<12	156 (27.6%)	154 (28.3%)
Unknown	14 (2.4%)	13 (2.4%)
LDH (U/L)		
Normal	406 (71.8%)	365 (67.1%)
Elevated	145 (25.7%)	167 (30.7%)
Unknown	14 (2.5%)	12 (2.2%)
β2-MG (mg/L)		
Normal	389 (68.8%)	353 (64.9%)
Elevated	164 (29.0%)	181 (33.3%)
Unknown	12 (2.2%)	10 (1.8%)
FLIPI-1		
Low risk	124 (21.9%)	107 (19.6%)
Intermediate risk	212 (37.5%)	202 (37.1%)
High risk	215 (38.0%)	222 (40.9%)
Unknown	14 (2.6%)	13 (2.4%)
FLIPI-2		
Low risk	161 (28.5%)	127 (23.3%)
Intermediate risk	279 (49.4%)	277 (50.8%)
High risk	111 (19.6%)	126 (23.3%)
Unknown	14 (2.5%)	14 (2.6%)
PRIMA-PI		
Low risk	265 (46.9%)	222 (40.7%)
Intermediate risk	125 (22.1%)	133 (24.4%)
High risk	162 (28.7%)	179 (33.0%)
Unknown	13 (2.3%)	10 (1.9%)
GELF criteria		
No GELF criteria	273 (48.3%)	193 (35.4%)
≥1 GELF criteria	262 (46.4%)	324 (59.6%)
Unknown	30 (5.3%)	27 (5.0%)

FL, follicular lymphoma; ECOG PS, Eastern Cooperative Oncology Group Performance Status; LDH, lactate dehydrogenase; β2-MG, β2 microglobulin; FLIPI-1, Follicular Lymphoma International Prognostic Index-1; FLIPI-2, Follicular Lymphoma International Prognostic Index-2; PRIMA-PI, PRIMA-prognostic index; GELF, Group d’Etude des Lymphomes Folliculaires.

### Treatment patterns

**Figure 2:**
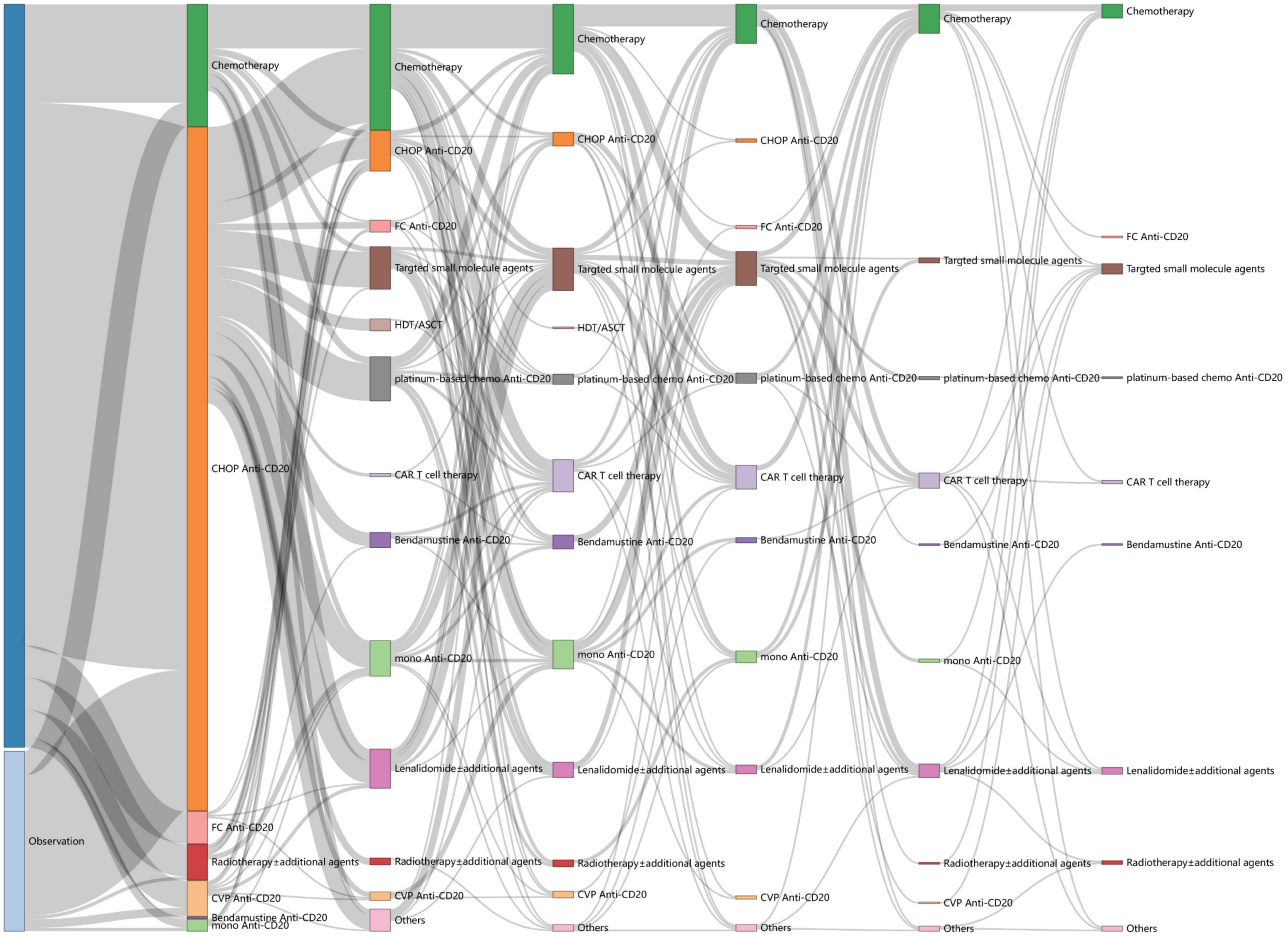
Sankey plot of treatment patterns across lines of therapy.

Treatment patterns across continuous lines (from the first to the sixth line) are illustrated in **Figure 2**. IM was the predominant choice for both the first and second lines of treatment, accounting for 81.5% (444/545) at the first line and 31.3% (75/240) at the second line, respectively. Anti-CD20 CHOP-like was the most frequently used regimen at first line (73.8%, 402/545), and 169 patients took anti-CD20 maintenance, with a median cycle of 6 (range: 1–12). The most frequently used IM regimen at the second line was anti-CD20 platinum-based regimens (e.g., DICE, GEMOX, GDP), accounting for 10.8% (26/240), with seven patients of them taking high-dose therapy/autologous stem cell transplantation (HDT/ASCT) for consolidation.

Nevertheless, the treatment options beyond the second line were characterized by great diversity and heterogeneity (**Supplementary Figure S1**). Chemotherapy alone (32.5%, 41/146), including platinum-based regimens, CHOP-like, CVP-like, bendamustine, and fludarabine-based regimens, was the most common third line of treatment, followed by IM (18.6%, 26/146), targeted small-molecule inhibitors (17.1%, 25/146) and CAR T-cell therapies (13.0%, 19/146). The other third line of treatments comprised anti-CD20 monotherapy (11.6%, 17/146), lenalidomide ± additional agents (6.8%, 9/146), radiotherapy ± additional agents (2.7%, 4/146), HDT/ASCT (0.7%, 1/146), and others (2.7%, 4/146). Targeted small-molecule inhibitors encompassed PI3K inhibitor, EZH2 inhibitor, BCL2 inhibitor, BTK inhibitor, and SYK inhibitor. The proportion of novel therapies in the fourth or later lines exceeded 40.0%, consisting of lenalidomide ± additional agents, CAR T-cell therapies, and targeted small-molecule inhibitors (44.3% at the fourth line, 42.6% at the fifth line, 42.9% at the sixth line).

### Treatment outcomes

**Figure 3:**
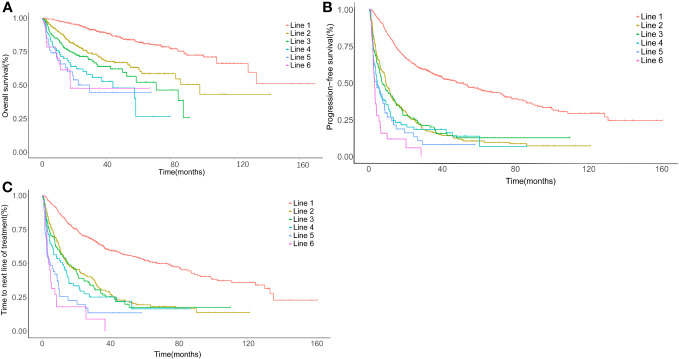
Time-to-event survival curves by lines of therapy. **(A)** Kaplan–Meier curve for overall survival (OS) calculated in each line, using the date of treatment initiation as the startpoint and the date of last follow-up or death as the endpoint. **(B)** Kaplan–Meier curve for progression-free survival (PFS) calculated in each line, using the date of treatment initiation as the startpoint and the date of progression or death as the endpoint. **(C)** Kaplan–Meier curve for time to next line of treatment (TTNLT) calculated in each line, using the date of treatment initiation as the startpoint and the date of next line of treatment or death as the endpoint. Red, first line; yellow, second line; green, third line; cyan, fourth line; blue, fifth line; rose, sixth line.

The ORR gradually decreased from 86.6% (first line) to 48.6% (third line), 40.4% (fifth line), and 31.4% (sixth line) (**Table 2**). A total of 566 patients were enrolled in the prognostic analyses by lines of treatment, with the median follow-up from diagnosis was 66.5 months (range: 1.4–212.0). The median OS after the first line was not reached, with a 5-year OS rate of 85.9%. Meanwhile, the median OS decreased to 36.9 months (95% CI: 18.0–55.7) at the fifth line of treatment (**Figure 3A**, **Table 3**). Additionally, a shorter disease-free interval was observed as more lines of therapies were administered (**Table 3**). The median PFS at the first line was 51.7 months (95% CI: 41.2–62.2). However, the median PFS declined to 4.1 months (95% CI: 0.3–7.9), and the median TTNLT decreased to 5.0 months (95% CI: 0.9–9.2) at the fifth line. Since there was a lack of durable response in later lines, the survival curves of PFS were close to one another in the fourth line to the sixth line, which was also seen in TTNLT (**Figures 3B, C**).

**Table 2:** Therapeutic efficacy of each line.

	First line (*n* = 545)	Second line (*n* = 240)	Third line (*n* = 146)	Fourth line (*n* = 88)	Fifth line (*n* = 47)	Sixth line (*n* = 28)
ORR % (*n*)	86.6% (471)	57.1% (137)	48.6% (71)	42.0% (37)	40.4% (19)	31.4% (9)
CR rate % (*n*)	54.8% (298)	26.7% (64)	17.8% (26)	17.0% (15)	14.9% (7)	7.1% (2)
PR rate % (*n*)	31.8% (173)	30.4% (73)	30.8% (41)	25.0% (22)	25.5% (12)	25.0% (7)
SD rate % (*n*)	4.0% (22)	18.3% (44)	17.1% (25)	15.9% (14)	21.3% (10)	7.1% (2)
PD rate % (*n*)	7.0% (38)	24.2% (58)	33.6% (49)	35.3% (31)	36.2% (17)	60.8% (17)
NA *n* (%)	2.6% (14)	0.4% (1)	0.7% (1)	1.1% (1)	–	–

ORR, objective response rate; CR, complete response; PR, partial response; SD, stable disease; PD, progressive disease; NA, not available.

**Table 3:** Long-term outcome of each line.

	First line (*n* = 545)	Second line (*n* = 240)	Third line (*n* = 146)	Fourth line (*n* = 88)	Fifth line (*n* = 47)	Sixth line (*n* = 28)
Median OS (95% CI)	NR	122.7 (72.2–173.2)	88.8 (53.1–124.5)	72.0 (25.7–118.2)	36.9 (18.0–55.7)	21.7 (15.6–57.9)
Median PFS (95% CI)	51.7 (41.2–62.2)	9.3 (7.7–10.9)	7.1 (4.8–9.5)	4.4 (2.5–6.3)	4.1 (0.3–7.9)	2.8 (2.1–3.5)
Median TTNLT (95% CI)	73.3 (57.2–89.4)	20.1 (12.3–27.8)	15.0 (9.1–20.9)	11.8 (5.6–18.2)	5.0 (0.9–9.2)	4.5 (2.9–6.1)

OS, overall survival; PFS, progression-free survival; TTNLT, time to next line of treatment; CI, confidence interval; NR, not reached.

The ORRs of various treatments at each line were further analyzed. The ORR decreased from 70.8% (first line) to 17.6% (fifth line) in the chemotherapy alone group and from 88.3% (first line) to 25.0% (fifth line) in the IM group (**Table 4**). In contrast, the reduction of ORR was less pronounced in patients treated with targeted small-molecule agents and CAR T-cell therapy. The ORR for CAR T-cell therapy varied among different lines of treatments, ranging from 85.7% (12/14) at the fourth line to 100.0% (9/9) at the fifth line. The ORR for targeted small-molecule agents ranged from 30.0% (6/20) at the fourth line to 56.0% (14/25) at the second line.

**Table 4:** ORR of various treatments at each line.

Classification	First line (%, *n*/*N*)	Second line (%, *n*/*N*)	Third line (%, *n*/*N*)	Fourth line (%, *n*/*N*)	Fifth line (%, *n*/*N*)
Chemotherapy only	70.8% (51/72)	41.9% (31/74)	22.0% (9/41)	21.7% (5/23)	17.6% (3/17)
Immunochemotherapy	88.3% (392/444)	71.8% (51/71)	69.2% (18/26)	40.0% (6/15)	25.0% (1/4)
Anti-CD20 mono	100.0% (7/7)	42.9% (9/21)	52.9% (9/17)	86.7% (6/7)	0.0% (0/2)
Lenalidomide ± additional agents	–	73.9% (17/23)	33.3% (3/9)	0.0% (0/5)	62.5% (5/8)
Radiotherapy ± additional agents	95.2% (20/21)	100.0% (4/4)	100.0% (4/4)	–	–
Targeted small molecule agents	–	56.0% (14/25)	40.0% (10/25)	30.0% (6/20)	33.3% (1/3)
HDT/ASCT	–	100.0% (7/7)	0.0% (0/1)	–	–
CAR T-cell therapy	–	100.0% (2/2)	94.3% (18/21)	85.7% (12/14)	100.0% (9/9)

ORR, overall response rate; HDT/ASCT, high-dose therapy and autologous stem cell transplantation; CAR T-cell therapy, chimeric antigen receptor T-cell therapy.

In terms of different treatment regimens, the ORR of IM was higher than that in the chemotherapy alone group at the second line (71.8% vs. 41.9%, *p* < 0.001), and the 5-year OS of IM was better than that of chemotherapy alone (79.0% vs. 50.3%, *p* = 0.0021). HDT/ASCT showed better 2-year PFS compared with IM (66.7% vs. 27.2%, *p* = 0.0034, **Supplementary Table S1**), and there was no significant difference in OS. At the third line, both 2-year PFS and 5-year OS of IM were still superior to chemotherapy alone (2y-PFS: 33.6% vs. 10.6%, *p* = 0.0077; 5y-OS: 65.5% vs. 38.2%, *p* = 0.018). Meanwhile, the CAR T-cell therapy showed PFS benefit over IM at the third line (2y-PFS: 58.9% vs. 33.6%, *p* = 0.024, **Supplementary Table S2**), which was also seen at the fourth line (2y-PFS: 64.3% vs. 13.5%, *p* = 0.023, **Supplementary Table S3**).

### Clinical trials

**Figure 4:**
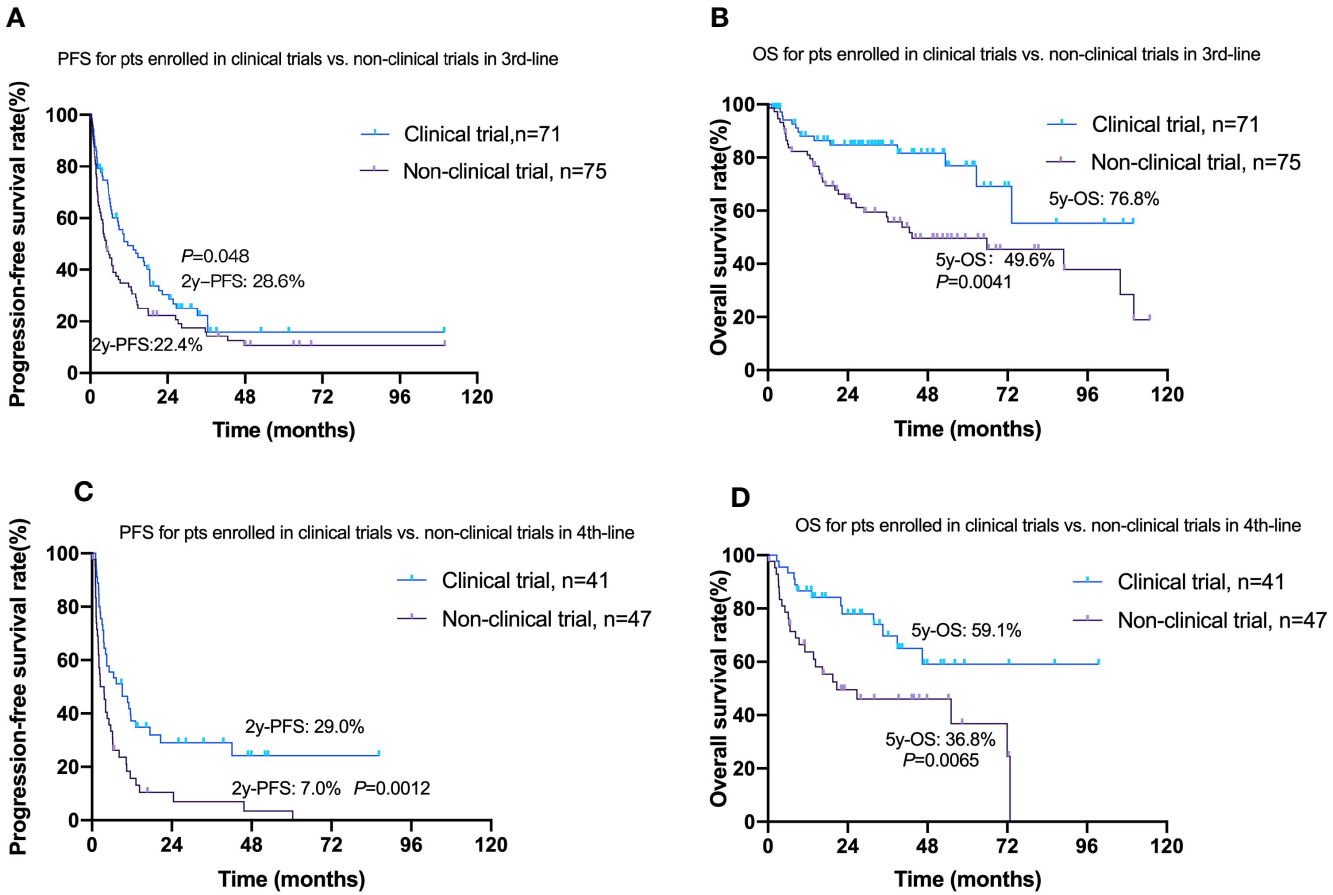
Comparison of survival curves for OS and PFS according to a history of enrollment in clinical trials at the third and fourth lines of treatment. **(A)** Progression-free survival (PFS) between the clinical trial group and nonclinical trial group at the third line of treatment. **(B)** Overall survival (OS) between the clinical trial group and nonclinical trial group at the third line of treatment. **(C)** PFS between the clinical trial group and nonclinical trial group at the fourth line of treatment. **(D)** OS between the clinical trial group and nonclinical trial group at the fourth line of treatment.

A multitude of new drugs with diverse mechanisms of action have been evaluated in clinical trials for the treatment of patients with FL. A total of 213 cases participated, spanning different categories including targeted small-molecule inhibitors (*n* = 79), anti-CD20 monotherapy (*n* = 37), CAR T-cell therapy (*n* = 43), IM regimens (*n* = 13), lenalidomide ± additional agents (*n* = 12), and chemotherapy alone (*n* = 12, **Supplementary Table S4**). Participation in clinical trials was primarily from the third-line treatment onwards, with 48.6% and 46.6% of the cases in the third-line and fourth-line treatment, respectively. The ORR in the clinical trial group was 56.3% at the third line and 55.6% at the fourth line, which were significantly higher than that in the nonclinical trial group (39.2% at the third line and 23.8% at the fourth line), with statistically significant differences (*p* < 0.05, **Supplementary Table S5**). Moreover, the clinical trial group demonstrated improvements in OS and PFS versus the nonclinical trial group at both the third line and the fourth line (**Figure 4**).

### POD24

A total of 133 patients (24.4%) relapsed within 24 months of first-line immunochemotherapy. Among them, 101 patients received first-line care in our facility, and the remaining 32 patients were initially treated elsewhere. The 5-year survival rate for the POD24 group was 70.4%, markedly lower compared to the non-POD24 group exhibiting a survival rate of 97.2% (HR: 9.3, 95% CI: 5.1–16.9, *p* < 0.001). Among patients undergoing third line and beyond, most of them experienced POD24, resulting in comparatively unfavorable outcomes versus the non-POD24 group (**Supplementary Tables S6**, **S7**). The ORR at the second line of chemotherapy alone, IM, lenalidomide ± additional agents, and HDT/ASCT in the POD24 group was 30.8%, 53.1%, 53.8%, and 100.0%, respectively. Notably, IM demonstrated a significant survival benefit as a subsequent therapy when compared with chemotherapy alone (*p* = 0.038, **Supplementary Table S8**). Although the survival curves for the HDT/ASCT and IM groups exhibited apparent differences, no statistically significant difference between the two groups was observed (*p* = 0.087). This is also observed when comparing the lenalidomide ± additional agents and IM group (*p* = 0.13, **Supplementary Figure S2**). A reasonable explanation for this could be the small sample size.

## Discussion

The present study retrospectively reviewed different treatment regimens and outcomes among Chinese FL patients across multiple lines of therapies. Despite being a retrospective single-center study, it incorporated various routine treatments and clinical trials in recent years. While the survival time was prolonged, the PFS and TTNLT decreased with increasing lines of treatments, which is consistent with the results from previous studies (20–25). The efficacy of treatment options in each line was further analyzed to explore optimal strategies for R/R FL patients. These findings provide a reference for constructing historical control cohorts for single-arm clinical trials as well as future studies in R/R FL.

There is considerable heterogeneity in treatment approaches at the third line and subsequent lines. When compared to previous cohorts in the USA and Europe, our cohort showed a higher proportion of chemotherapy alone (28% at the third line) compared to 10% in the SCHOLAR-5 study and 11.2% in the ReCORD-FL study. In contrast, HDT/ASCT was utilized less in our cohort (only one patient at the third line, 0.7%), compared with 13% in the LEO-CReWE study and 23% in the SCHOLAR-5 study. PI3K inhibitors, lenalidomide, and CAR T-cell therapy were administrated in both our study and the LEO-CReWE study at the third line and subsequent lines. Since the SCHOLAR-5 study was designed in part to create an external control group for the ZUMA-5 trial, which examined the clinical effectiveness of axicabtagene ciloleucel, CAR T-cell therapies were not covered in the SCHOLAR-5 study (21). The variations in treatment patterns among cohorts can be attributed to geographical and population characteristics. Moreover, the accessibility and policy of novel drugs during the study period were also related to these discrepancies, which will be discussed further in this paper.

The ORR in our study at the third line was 48.6%, which was lower than that of SCHOLAR-5 (68.3%) and LEO-CReWE (69.5%) and was relatively similar to a Japanese cohort (53.7%) and a meta-analysis (58.5%) (21–23, 25). However, it is important to note that there were variations in population, time spans, and inclusion and exclusion criteria among these studies. Therefore, comparisons should be made with caution. Regarding the survival outcomes, the median OS in our cohort, SCHOLAR-5, and ReCORD-FL were 88.3, 67.6, and 133.7 months, respectively, which indicated the indolent nature of FL. Despite advances in novel therapeutic strategies, the prognosis for patients who underwent multiple lines of treatment remained poor. The median PFS fell below 18 months (range, 7.1–17) after the third line and decreased to less than 12 months (range, 4.4–12.0) after the fourth line. These findings highlighted the progressive invasiveness and drug resistance growing with increasing numbers of relapses.

Although IM demonstrated superiority over chemotherapy alone at the third line, the outcomes were still suboptimal. Fortunately, the development of new drugs in recent years has broadened the treatment options for R/R FL. Several studies have suggested that novel agents exhibited promising antilymphoma activities (5, 10, 16, 26, 27). The ELARA and ZUMA-5 studies demonstrated high ORR and durable remission with tisagenlecleucel and axicabtagene ciloleucel (CD19 CAR T-cell therapies), with ORRs of 86.2% and 94%, respectively (10, 11). Similar findings were observed in our study, but the unique toxicities such as cytokine release syndrome (CRS) and immune effector cell-associated neurotoxicity syndrome (ICANS), limited accessibility (complex production process, restricted in specific hospital and continual assessment after medication), and high costs have impeded their further clinical application. CD20/CD3 bispecific antibodies have a similar mechanism with CAR T-cell therapy. Previous studies indicated the bispecific antibodies achieved a similar ORR ranging from 78.9% to 90.0% while demonstrating lower incidences of adverse events, including CRS and ICANS. Furthermore, bispecific antibodies were also more convenient for clinical applications (27, 28). Meanwhile, combination therapy with CD20/CD3 bispecific antibodies and lenalidomide has shown improved remission rates, making it a more promising and practical approach for managing R/R FL patients (29).

In the third line and subsequent lines, small-molecule inhibitors such as EZH2 and PI3K inhibitors have exhibited certain antitumor activities when administered as monotherapy. The ORR varied from 35% to 65%, and the median PFS approached 1 year. Currently, there is ongoing exploration of combination therapies that incorporate these inhibitors with other agents to improve efficacy (5). Based on the aforementioned discussions, the selection of individualized treatment regimens should carefully consider the balance between patient condition, tumor burden, drug toxicities, and quality of life.

Given the difficulty in obtaining novel drugs available in the market during the study period, CAR T-cell therapies, small-molecule inhibitors, and most of the lenalidomide-based regimens were administered in clinical trials. Our findings revealed that, compared with standard care, participating in clinical trials could result in higher remission rates and improved survival benefits in later lines. Collectively, these findings indicated the current landscape of multiple clinical trials for R/R FL and the high enrollment rates of clinical trials in our center. However, most of the therapeutic efficacies for these novel agents were primarily based on results of single-armed phase 2 clinical trials with relatively short follow-up periods. Consequently, expanding the sample size and extending the follow-up time might be the key aims of future clinical trials.

The majority of patients in the later lines were found to have POD24, which is consistent with previous research reports (22, 23). This high proportion of POD24 patients might contribute to the low remission rate of this cohort. However, the 5-year OS rate was 70.4% in our cohort, which was consistent with other studies in the past 2 years and exhibited an improvement over the previous study reported in 2015 (6, 7). It might be related to improved treatment modalities and increased attention to this population. These findings highlight the poorer response rates and prognosis of the POD24 group in later lines of treatment compared to the non-POD24 group.

Currently, there are no standard options for patients with early relapse after initial treatments, making the POD24 group a suitable and recommended population for clinical trials. For patients inappropriate for clinical studies, HDT/ASCT and lenalidomide ± additional agents have shown promising durable remission as a second line of treatment. It has been reported that outcomes in patients who received HDT/ASCT were superior to those in nontransplantation patients at the second-line and third-line therapies (30–32). However, most of these studies were published prior to the introduction of the concept of POD24 in 2015. In an article published by Smith et al. in 2018, it was demonstrated that undergoing HDT/ASCT resulted in a promising 5-year OS rate of 70% (33) in the POD24 group. Despite these findings, HDT/ASCT was not commonly used for treating R/R FL patients in our center due to concerns about the toxicities associated with high-dose chemotherapy. Moreover, novel therapies have significantly impacted the position and timing of SCT. According to two previous studies, the ORR of R2 regimens in the POD24 group was reported as 65% and 80%, showing a relatively small difference compared to the non-POD24 group (13, 34). The GALEN study, which evaluated the combination of obinutuzumab and lenalidomide for R/R FL patients, showed no difference in outcomes between POD24 and non-POD24 patients (35). Further investigation is warranted to determine the optimal subsequent therapy for POD24 patients.

The strengths of this paper were comprehensive treatment regimens and detailed analysis. However, several limitations exist in this study. Firstly, owing to the retrospective and single-center nature of the study, there is a possibility of selection bias. Secondly, the sample size of our cohort in the fourth line and subsequent lines was relatively small, and the diversity of treatment options limited the comparability of the intervention response at each line of therapy. Thirdly, in order to reflect real clinical conditions, patients who developed histopathological transformation (HT) during the course of treatment were not excluded. Nonetheless, since HT is associated with poor clinical outcomes, there may be a disparity in prognosis between patients with HT and those without.

In conclusion, FL presents a wide heterogeneity in its indolent clinical course, which necessitates multiple therapy attempts. By scrutinizing the complex treatment patterns and the effectiveness of each treatment line, these findings demonstrated the poor outcomes in R/R FL patients, especially in the POD24 group, with a worsening impact on key indicators such as ORR, PFS, and OS as patients experienced multiple relapses. These results emphasized the importance of identifying predictive markers and developing novel treatment approaches for R/R FL patients. Furthermore, our study provides valuable benchmarks that can be used for comparisons in future clinical trials.

## Data availability statement

The data analyzed in this study is subject to the following licenses/restrictions: The raw data of this article will be made available by the authors, without undue reservation. Requests to access these datasets should be directed to songyuqin622@outlook.com.

## Ethics statement

The study was reviewed and approved by the Ethical Review Committee of Peking University Cancer Hospital. Informed consent was obtained from all individual participants included in the study. The studies were conducted in accordance with the local legislation and institutional requirements. Written informed consent for participation was not required from the participants or the participants’ legal guardians/next of kin in accordance with the national legislation and institutional requirements.

## Author contributions

JL: Data curation, Formal Analysis, Writing – original draft. LZ: Data curation, Writing – review & editing. YH: Validation, Visualization, Resources, Writing – review & editing. RN: Data curation, Writing – review & editing. YL: Data curation, Writing – review & editing. HY: Data curation, Writing – review & editing. YY: Data curation, Writing – review & editing. DW: Data curation, Writing – review & editing. YT: Data curation, Writing – review & editing. FF: Data curation, Writing – review & editing. WL: Data curation, Writing – review & editing, Conceptualization, Investigation, Supervision, Validation. JZ: Conceptualization, Investigation, Supervision, Writing – review & editing, Funding acquisition, Project administration, Resources. LP: Investigation, Project administration, Resources, Supervision, Writing – review & editing, Data curation. YS: Investigation, Project administration, Resources, Supervision, Writing – review & editing, Software, Validation, Visualization.
