# Supplementary material for: Management and clinical outcomes of follicular lymphoma across continuous lines of treatments: a retrospective analysis in China
Source: Front Oncol. 2023 Oct 24;13:1264723. doi: 10.3389/fonc.2023.1264723 (PMC10628462; doi:10.3389/fonc.2023.1264723)
Supplement: Supplementary file 1 [file DataSheet_1.docx]

Supplementary Material

**Supplementary TABLE S1** Comparison on OS and PFS at the 2nd-line of treatment.

|  | N | PFS | | OS | | |
| --- | --- | --- | --- | --- | --- | --- |
|  |  | 2-year(95%CI) | HR (95%CI) | 2-year(95%CI) 5-year(95%CI) | | HR (95%CI) |
| Chemotherapy alone | 74 | 19.3%(9.4%-29.2%) | 1.279(0.899-1.819) | 90.3%(80.8%-96.1%) | 50.3%(36.5%-61.5%) | 2.686(1.588-4.543) |
| IM | 75 | 27.2%(16.6%-38.9%) | 1 | 92.6%(86.2%-97.9%) | 79.0%(66.9%-86.9%) | 1 |
| Anti-CD20 monotherapy | 21 | 24.8%(10.8%-48.5%) | 0.888(0.525-1.512) | 100.0% | 89.2%(63.0%-97.2%) | 0.730(0.271-1.968) |
| Lenalidomide ± additional agents | 23 | 31.7%(14.5%-45.7%) | 0.698(0.424-1.148) | 95.5%(71.5%-99.2%) | 85.6%(61.7%-95.1%) | 0.694(0.260-1.857) |
| Small molecule inhibitors | 25 | 8.0%(1.4%-22.5%) | 1.329(0.912-2.232) | 84.0%(62.8%-93.7%) | 84.0%(62.8%-93.7%) | 0.854(0.302-2.415) |
| HDT/ASCT | 7 | 66.7%(31.6%-80.5%) | 0.247(0.114-0.535) | 90.4%(80.8%-95.2%) | 83.3%(33.3%-97.4%) | 0.605(0.119-3.076) |
| Others | 18 | 27.5%(12.6%-43.9%) | 1.447(0.712-2.939) | 100.0% | 75.0% | 0.340(0.924-1.250) |

PFS, progression-free survival; OS, overall survival; HR, hazard ratio; CI, confidence interval; IM, Immunochemotherapy; HDT/ASCT, high-dose chemotherapy followed by autologous stem cell transplantation;

**Supplementary TABLE S2** Comparison on OS and PFS at the 3rd-line of treatment.

|  | N | PFS | | OS | | |
| --- | --- | --- | --- | --- | --- | --- |
|  |  | 2-year(95%CI) | HR (95%CI) | 2-year(95%CI) 5-year(95%CI) | | HR (95%CI) |
| Chemotherapy alone | 41 | 10.6%(3.4%-22.5%) | 2.033(1.215-3.403) | 51.5%(36.5%-65.1%) | 38.2%(23.6%-53.7%) | 2.421(1.237-4.740) |
| IM | 26 | 33.6%(16.3%-51.8%) | 1 | 84.2%(63.3%-94.1%) | 65.5%(39.5%-82.5%) | 1 |
| Anti-CD20 monotherapy | 17 | 20.6%(7.3%-44.9%) | 1.343(0.673-2.679) | 88.2%(60.6%-96.6%) | 65.3%(28.5%-86.5%) | 0.827(0.257-2.657) |
| Small molecule inhibitors | 25 | 9.3% | 1.746(0.928-3.285) | 87.3%(63.5%-95.7%) | 87.3% | 0.586(0.175-1.968) |
| CAR T cell therapy | 21 | 58.9%(32.3%-78.0%) | 0.477(0.230-0.988) | 82.2%(54.3%-93.9%) | 54.8%(9.1%-86.0%) | 0.881(0.271-2.858) |

PFS, progression-free survival; OS, overall survival; HR, hazard ratio; CI, confidence interval; IM, Immunochemotherapy; CAR T cell therapy, chimeric antigen receptor T cell therapy;

**Supplementary TABLE S3** Comparison on OS and PFS at the 4th-line of treatment.

|  | N | PFS | | OS | | |
| --- | --- | --- | --- | --- | --- | --- |
|  |  | 2-year(95%CI) | HR (95%CI) | 2-year(95%CI) 5-year(95%CI) | | HR (95%CI) |
| Chemotherapy alone | 23 | 4.3%(0.0%-18.2%) | 1.432(0.772-2.838) | 43.5%(23.3%-61.1%) | 37.3%(17.5%-57.0%) | 1.454(0.620-3.406) |
| IM | 15 | 13.5%(1.2%-41.0%) | 1 | 62.2%(31.0%-82.5%) | 33.2%(6.4%-64.2%) | 1 |
| Small molecule inhibitors | 20 | 5.0%(0.4%-20.5%) | 1.272(0.617-2.624) | 78.9%(22.8%-91.0%) | 65.8%(31.3%-85.9%) | 0.502(0.159-1.584) |
| CAR T cell therapy | 14 | 64.3%(1.4%-30.4%) | 0.317(0.117-0.858) | 75.0%(39.4%-91.5%) | 62.5%(26.2%-84.8%) | 0.632(0.194-2.064) |

OS, overall survival; PFS, progression-free survival; IM, Immunochemotherapy; CAR T cell therapy, chimeric antigen receptor T cell therapy; HR, hazard ratio; CI, confidence interval;

**Supplementary TABLE S4** Participation in clinical trials in each line of treatment.

| N | 1st-line(n=544) | 2nd-line(n=240) | 3rd-line(n=146) | 4th-line(n=88) | 5th-line(n=47) | 6th-line(n=28) |
| --- | --- | --- | --- | --- | --- | --- |
|  | Yes No | Yes No | Yes No | Yes No | Yes No | Yes No |
| Chemotherapy alone | 0 72 | 9 65 | 3 38 | 0 23 | 0 17 | 0 8 |
| IM | 2 442 | 5 66 | 4 22 | 2 13 | 0 4 | 0 3 |
| Anti-CD20 monotherapy | 0 7 | 15 6 | 15 2 | 5 2 | 2 0 | 0 0 |
| Lenalidomide ± additional agents | 0 0 | 10 13 | 2 7 | 0 5 | 0 8 | 0 4 |
| Radiotherapy±additional agents | 0 21 | 0 4 | 0 4 | 0 0 | 0 1 | 0 2 |
| CAR T cell therapy | 0 0 | 2 0 | 18 1 | 14 0 | 8 1 | 1 1 |
| Small molecule inhibitors | 0 0 | 25 0 | 25 0 | 20 0 | 3 0 | 6 0 |
| HDT/ASCT | 0 0 | 0 7 | 0 1 | 0 0 | 0 0 | 0 0 |
| Others | 0 0 | 9 4 | 4 0 | 0 4 | 2 1 | 2 1 |
| Total (n, %) | 2 542  (0.4%) (99.6%) | 75 165  (31.2%) (68.8%) | 71 75  (48.6%) (51.4%) | 41 47  (46.6%) (53.4%) | 15 32  (31.9%) (68.1%) | 9 19  (32.2%) (67.8%) |

IM, Immunochemotherapy; CAR T cell therapy, chimeric antigen receptor T cell therapy; HDT/ASCT, high-dose chemotherapy followed by autologous stem cell transplantation;

**Supplementary TABLE S5** Comparison of ORR between clinical trial group and non-clinical trial group in 2nd-line to 5th-line.

|  | 2nd-line  N ORR *P* value | 3rd-line  N ORR *P* value | 4th-line  N ORR *P* value | 5th-line  N ORR *P* value |
| --- | --- | --- | --- | --- |
| Clinical trials | 75 60.0% 0.58 | 71 56.3% 0.047 | 41 55.6% 0.004 | 15 60.0% 0.12 |
| Non-clinical trials | 165 40.0% | 75 39.2% | 47 23.8% | 32 34.4% |

ORR, overall response rate;

**Supplementary TABLE S6** Comparison of ORR between POD24 group and non-POD24 group in 2nd-line to 5th-line.

|  | 2nd-line  N ORR *P* value | 3rd-line  N ORR *P* value | 4th-line  N ORR *P* value | 5th-line  N ORR *P* value |
| --- | --- | --- | --- | --- |
| POD24 | 127 47.2% 0.004 | 82 50.0% >0.05 | 48 62.5% 0.77 | 27 37.9% 0.15 |
| Non-POD24 | 48 72.9% | 27 50.0% | 16 56.3% | 9 28.6% |

ORR, overall response rate; POD24: progressive disease or death due to progressive disease events in the 24 months initiating first-line immunochemotherapy;

**Supplementary TABLE S7** Comparison of outcomes between POD24 group and non-POD24 group in 2nd-line to 5th-line.

|  | 2nd-line  N 5y-OS *P* value | 3rd-line  N 5y-OS *P* value | 4th-line  N 5y-OS *P* value | 5th-line  N 5y-OS *P* value |
| --- | --- | --- | --- | --- |
| POD24 | 127 61.4% 0.002 | 82 55.0% 0.035 | 48 48.9% 0.36 | 27 48.2% 0.65 |
| Non-POD24 | 48 82.6% | 27 80.0% | 16 75.0% | 9 71.4% |

OS, overall survival; POD24: progressive disease or death due to progressive disease events in the 24 months initiating first-line immunochemotherapy;

**Supplementary TABLE S8** Comparison of ORR and survival rate of POD24 group by treatment classes at 2nd-line.

|  |  | clinical efficacy | | Long-term survival | | |
| --- | --- | --- | --- | --- | --- | --- |
|  | N | ORR | *P* value | 5y-OS (%) | HR(95%CI) | *P* value |
| Chemotherapy alone | 39 | 30.8%(12/39) | 0.09 | 46.1% | 2.16(1.08-4.31) | 0.038 |
| IM | 32 | 53.1%(17/32) | - | 58.2% | 1 | - |
| Anti-CD20 monotherapy | 9 | 11.1%(1/9) | 0.05 | 71.1% | 0.54(0.15-1.99) | 0.44 |
| Lenalidomide ± additional agents | 13 | 53.8%(7/13) | 1.00 | 81.1% | 0.41(0.12-1.38) | 0.29 |
| Small molecule inhibitors | 20 | 55.5%(11/20) | 1.00 | 80.0% | 0.43(0.19-1.01) | 0.13 |
| HDT/ASCT | 6 | 100.0%(6/6) | 0.63 | 66.7% | 0.21(0.07-0.61) | 0.087 |

ORR, overall response rate; POD24, progressive disease or death due to progressive disease events in the 24 months initiating first-line immunochemotherapy; IM, Immunochemotherapy; HDT/ASCT, high-dose chemotherapy followed by autologous stem cell transplantation; HR, hazard ratio; CI, confidence interval;

**Supplementary FIGURE S1** Proportion of various modalities in each line of treatment.


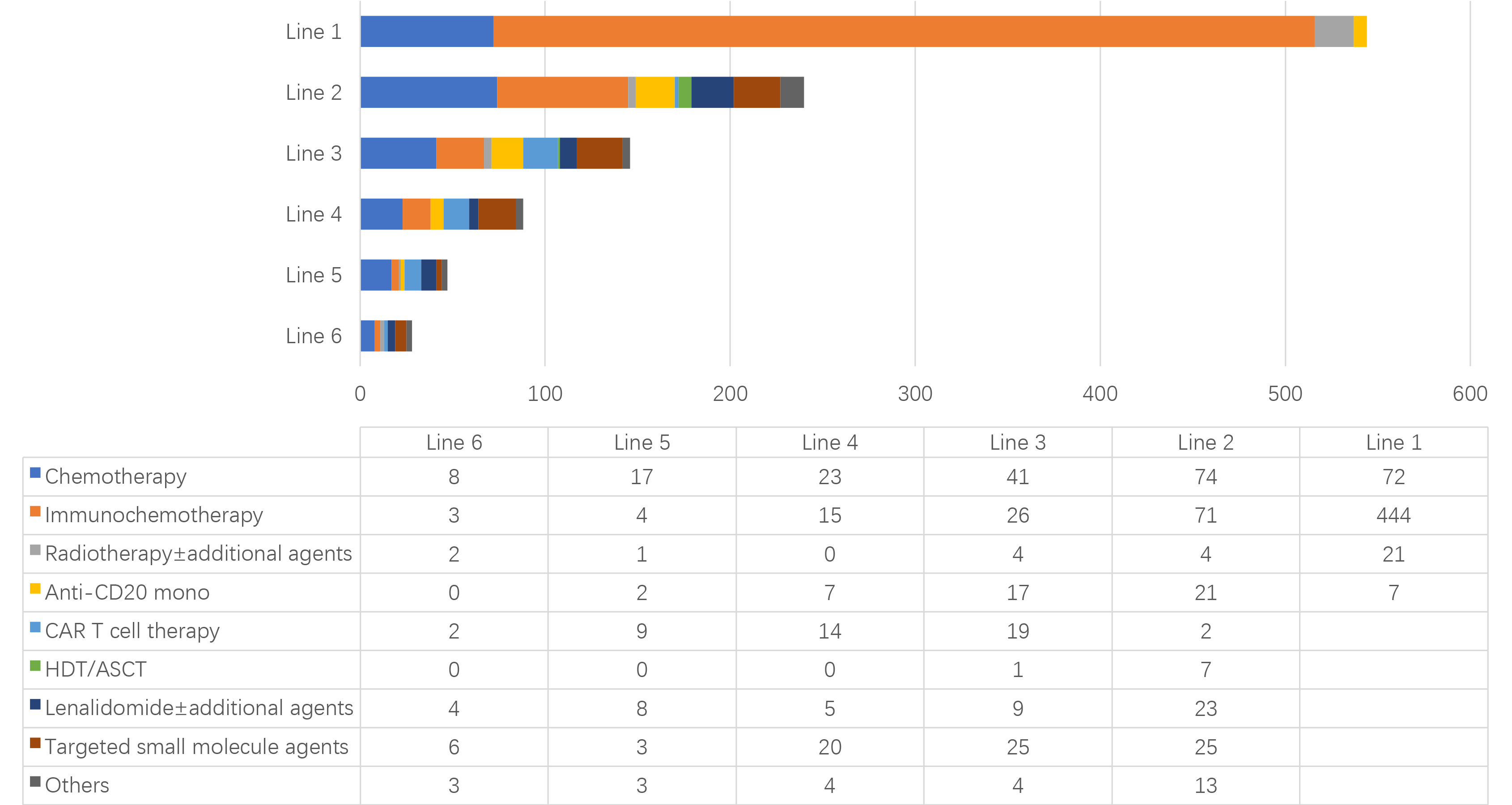


CAR T cell therapy, chimeric antigen receptor T cell therapy; HDT/ASCT, high-dose chemotherapy followed by autologous stem cell transplantation;

**Supplementary FIGURE S2** Survival curves between groups in 2nd-line of treatment for patients with POD24.

**
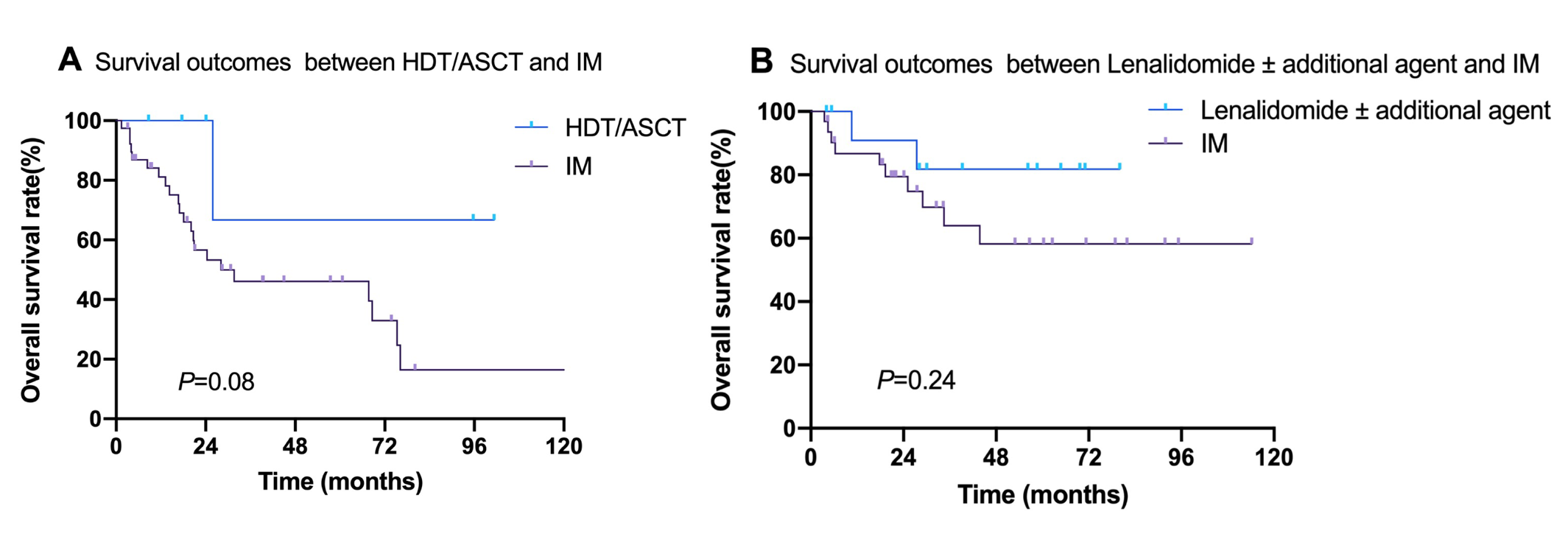
**

**(A)**Comparison of survival curves comparing patients treated with IM and HDT/ASCT. **(B)** Comparison of survival curves comparing patients treated with IM and lenalidomide ±additional agent. IM, Immunochemotherapy; HDT/ASCT, high-dose chemotherapy followed by autologous stem cell transplantation;
